# Root-associated Bacterial Communities of Sugarcane Cultivated for Traditional Wasanbon Sugar differ among Cultivation Sites in Kagawa, Japan

**DOI:** 10.1264/jsme2.ME26030

**Published:** 2026-09-04

**Authors:** Yasunobu Yamada, Md Shahidul Islam, Kaoru Matsumoto, Masahiro Morokuma, Mika Nomura

**Affiliations:** 1 Faculty of Agriculture, Kagawa University, 2393 Miki, Kita, Kagawa, 761–0795, Japan; 2 The United Graduate School of Agricultural Sciences, Ehime University, 790–8566, Japan; 3 Bangladesh Sugarcrop Research Institute, Joypurhat Substation, 5900, Bangladesh; 4 Bioinformatics Analysis Center, Kagawa University, 2393 Miki, Kita, Kagawa, 761–0795, Japan; 5 Faculty of Agriculture, Saga University, 1 Honjyo, Saga, 840–8502, Japan

**Keywords:** 16S rRNA gene sequencing, root-associated bacteria, sugarcane, wasanbon sugar

## Abstract

Sugarcane for traditional Wasanbon sugar has long been cultivated in Higashi-Kagawa. Three sugarcane cultivars were cultivated at three sites in Kagawa Prefecture, and root-associated bacterial communities were analyzed using 16S rRNA gene amplicon sequencing. Bacterial community compositions varied primarily according to the cultivation site rather than the cultivar, and soil bacterial communities showed similar site-dependent clustering patterns. These results indicate that the cultivation site affects the root-associated bacterial community structure.

Root-associated bacterial communities play important roles in plant growth, nutrient acquisition, and stress tolerance (Bulgarelli *et al.*, 2013). The host genotype and soil environment have both been reported to affect the composition of the root-associated microbiota. In sugarcane, the host genetic background was shown to affect the root bacterial community structure (Dong *et al.*, 2018). Studies on other plant systems demonstrated that soil properties and local environmental conditions were major factors affecting the composition of the microbial community (Edwards *et al.*, 2015). Sugarcane harbors diverse bacterial communities, including diazotrophic and plant growth–promoting bacteria that contribute to nutrient acquisition and plant productivity (Boddey *et al.*, 2003). In Higashi-Kagawa, Japan, sugarcane has long been cultivated for the production of traditional wasanbon sugar. This cultivation system has been maintained for centuries despite the region having a temperate climate that is generally considered to be suboptimal for sugarcane growth. Although the ecological factors supporting the long-term stability of this cultivation system remain unclear, one possibility is the impact of the local soil environment on sugarcane-associated microbial communities under these environmental conditions. However, the microbial communities associated with sugarcane grown in this traditional production region have yet to be characterized in detail. In the present study, we exami­ned the root-associated bacterial communities of sugarcane cultivated at different sites in Kagawa, Japan to establish whether the microbial community composition differs among cultivation sites. Three sugarcane cultivars were used in this study: *Saccharum officinarum*, *Saccharum sinense* provided by the National Agriculture and Food Research Organization (NARO), and a commercial cultivar derived from *S. sinense*. Plants were grown at three cultivation sites in Kagawa Prefecture: Higashi-Kagawa (a traditional wasanbon production site), the university farm, and Ikenobe (a fallow field site). All plants were planted as stem cuttings on March 31, 2023 and were sampled on August 30, 2023. Although plant biomass was not quantitatively measured in this study, no obvious differences in plant growth were noted among the cultivation sites during field observations at the time of sampling. After harvesting, roots were gently shaken to remove loosely attached soil particles and washed with sterile distilled water. The roots were then surface-sterilized with 3% sodium hypochlorite for 20 min and subsequently rinsed three times with sterile distilled water to remove residual disinfectant. This procedure was performed to minimize the presence of surface-associated microorganisms prior to DNA extraction. Surface-sterilized roots were then used for DNA extraction. Bacterial genomic DNA was extracted from sugarcane roots and soil samples using the DNeasy PowerSoil Pro Kit (QIAGEN) according to the method described by Supriadi *et al.* (2020). Bacterial community compositions were analyzed by sequencing the V3–V4 region of the 16S rRNA gene. After adapter trimming, sequences were processed using the DADA2 pipeline (Callahan *et al.*, 2016) to generate amplicon sequence variants (ASVs). ASVs assigned to chloroplasts or mitochondria were removed. In addition, low-prevalence and low-‍abundance ASVs were filtered prior to a downstream anal­ysis. The number of reads per sample after ASV filtering ranged from 6,473 to 37,830. Rarefaction curves indicated that sequencing depth was sufficient for all samples because the curves approached saturation (Fig. S1). A phylogenetic tree for the UniFrac anal­ysis was constructed from ASV sequences using DECIPHER (Wright, 2015) and phangorn (Schliep, 2011). Alpha diversity was calculated using observed ASV richness and the Shannon diversity index. Differences among cultivation sites were tested using generalized linear models (GLMs), with the university farm being used as the reference level. Beta diversity was evaluated using Bray-Curtis and weighted UniFrac distances. A‍ ‍permutational multivariate anal­ysis of variance (PERMANOVA) was conducted using root samples, with cultivation site and host cultivar being included in the model to assess their relative contributions to bacterial community compositions. To identify differentially abundant bacterial families between Higashi-Kagawa and the combined Ikenobe/university farm group, an ANCOM-BC2 anal­ysis was performed using the ANCOMBC R package (Lin and Peddada, 2024), with a binary site variable as the fixed effect, the Benjamini–Hochberg correction, and a prevalence cut-off of 20%. Taxa with q<0.05 that passed the sensitivity anal­ysis were considered to be significant.

The alpha diversity of root-associated bacterial communities differed among cultivation sites. Observed ASV richness and the Shannon diversity index were both significantly lower in Ikenobe than in the two other cultivation sites (Fig. S2). In contrast, no significant difference was observed in alpha diversity between Higashi-Kagawa and the university farm.

A beta diversity anal­ysis revealed the clear separation of root-associated bacterial communities according to the cultivation site (Fig. 1A and B). This pattern was observed using both Bray-Curtis (Fig. 1A) and weighted UniFrac distance metrics (Fig. 1B). Similar site-dependent separation was observed when root and soil bacterial communities were both analyzed together (Fig. 1C and D), indicating that bacterial community compositions differed among cultivation sites in both root-associated and soil environments. PERMANOVA (9,999 permutations) showed that the sampling location markedly affected the bacterial community composition (Bray–Curtis R^2^=0.63, *P*=1×10^–4^; weighted UniFrac R^2^=0.73, *P*=1×10^–4^), whereas the contribution of the host cultivar was weaker (Bray–Curtis R^2^=0.073, *P*=0.046; weighted UniFrac R^2^=0.066, *P*=0.012). These results indicate that the cultivation site had a stronger effect on root-associated bacterial communities than the host genotype in this study. Taxonomic composition at the class level also differed among cultivation sites (Fig. S3). Collectively, these results indicate that the root-associated bacterial communities of sugarcane were primarily affected by the soil environment of the cultivation site rather than by the host cultivar. Since many root-associated bacteria originate from surrounding soil microbial communities, differences in soil bacterial compositions among cultivation areas may be reflected in the root-associated microbiome. Previous studies demonstrated that sugarcane-associated bacterial communities may include diazotrophic and plant growth-promoting taxa (Boddey *et al.*, 2003; Carvalho *et al.*, 2014). Interestingly, the traditional production area in Higashi-Kagawa exhibited a distinct microbial community structure from the other cultivation sites exami­ned in this study. The ANCOM-BC2 anal­ysis revealed significant differences in sugarcane root-associated bacterial communities among cultivation sites, with the bacterial taxa enriched in Higashi-Kagawa being distinct from those in the university farm and Ikenobe sites (Table S1 and S2). In Higashi-Kagawa, several bacterial families were significantly enriched (Table‍ ‍S1). These taxa have frequently been associated with‍ ‍oligotrophic or acidic soil environments, the degradation of recalcitrant organic matter, and adaptation to stable‍ ‍soil environments. Therefore, the enrichment of *Ktedonobacteraceae*, *Solirubrobacteraceae*, *Acidobacteriaceae*, and *Bryobacteraceae* may reflect the characteristics of relatively stable cultivated soils because these taxa are commonly detected in oligotrophic, acidic, and less-disturbed environments characterized by slow organic matter turnover (Fierer *et al.*, 2007; Caro *et al.*, 2023). In addition, members of *Acetobacteraceae*, *Burkholderiaceae*, and *Beijerinckiaceae* include sugar-utilizing, nitrogen-fixing, and plant-associated bacteria previously reported in root-associated communities of sugarcane and other grasses (Carvalho *et al.*, 2014). These findings suggest that local soil environments in Higashi-Kagawa favor an oligotrophic, sugar-adapted, and potentially diazotrophic microbial assemblage. In contrast, distinct bacterial families were significantly enriched in the Ikenobe and university farm sites (Table S2). Highly enriched taxa, including *Fibrobacteraceae*, *Steroidobacteraceae*, and *Microscillaceae*, are commonly associated with the degradation of plant-derived poly­saccharides and carbon turnover in soil environments (Fierer‍ ‍*et al.*, 2007; Ransom-Jones *et al.*, 2012), whereas members‍ ‍of *Bacillaceae*, *Rhizobiaceae*, *Streptomycetaceae*, *Micromonosporaceae*, and *Pseudonocardiaceae* have frequently been associated with nitrogen metabolism, nutrient mobilization, phytohormone production, and biocontrol in root-associated environments (Hirsch and Valdés, 2010; Gopalakrishnan *et al.*, 2013). Collectively, these results indicate that the local soil environments in Higashi-Kagawa and site-specific environmental conditions in the Ikenobe and university farm sites differentially shaped the sugarcane root-associated bacterial communities, potentially contributing to distinct ecological strategies related to nutrient acquisition, stress adaptation, and plant health maintenance. However, the specific functional roles of these taxa in sugarcane have yet to be experimentally confirmed. Since this study included only one traditional Wasanbon cultivation site, broader comparisons across multiple traditional cultivation fields, together with functional anal­yses, will be necessary to establish whether these microbial characteristics are consistently associated with traditional Wasanbon cultivation.

## Accession numbers

Sequence data have been deposited in DDBJ under the following accession numbers: root-associated bacteria, DRR567452–DRR567472; soil rhizosphere, DRR567473–DRR567481.

## Citation

Yamada, Y., Islam, M. S., Matsumoto, K., Morokuma, M., and Nomura, M. (2026) Root-associated Bacterial Communities of Sugarcane Cultivated for Traditional Wasanbon Sugar differ among Cultivation Sites in Kagawa, Japan. *Microbes Environ ***41**: ME26030.

https://doi.org/10.1264/jsme2.ME26030

## Supplementary Material

Supplementary Material

## Figures and Tables

**Fig. 1.**
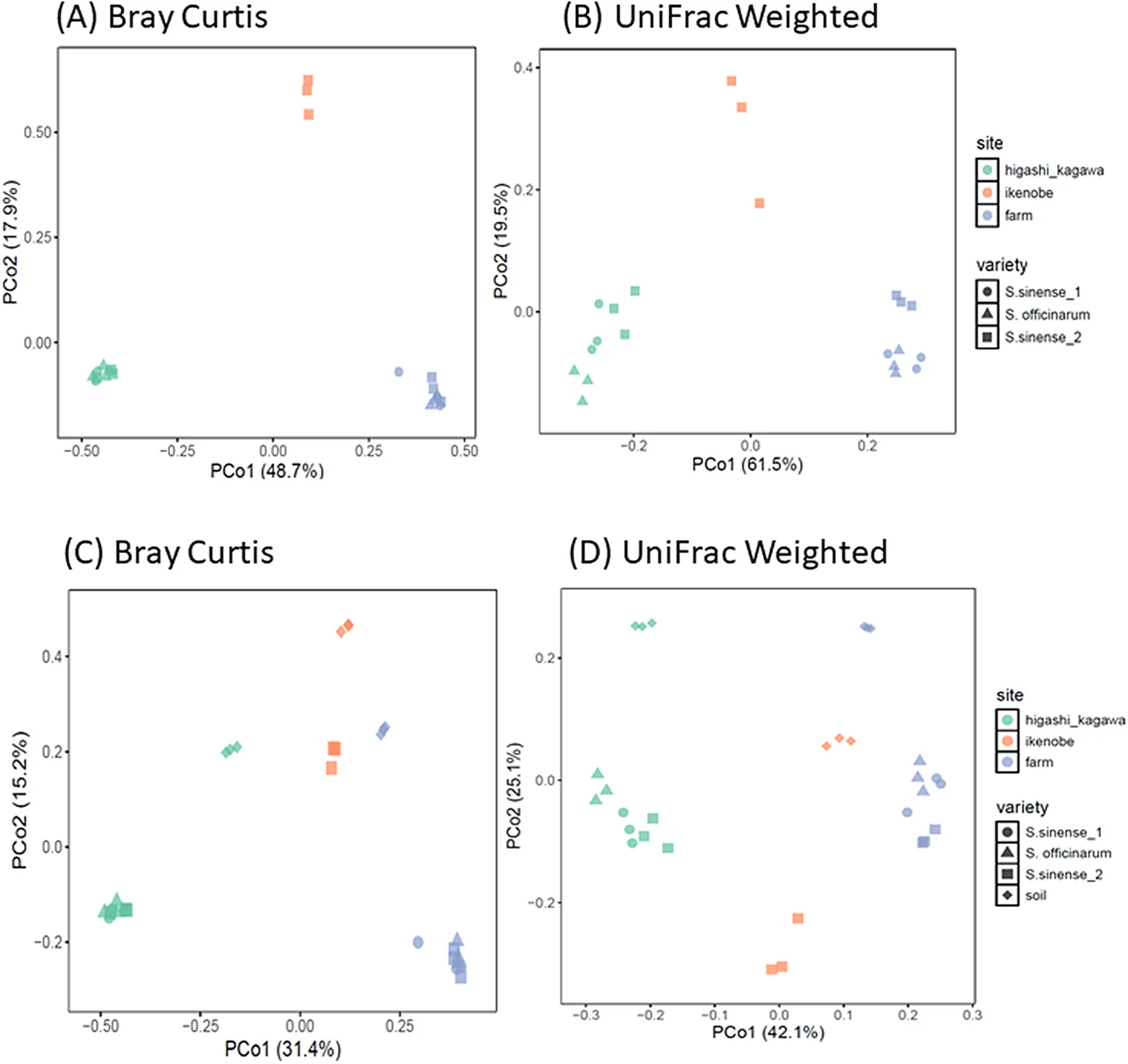
Principal coordinates anal­ysis (PCoA) of bacterial communities based on Bray–Curtis (A, C) and weighted UniFrac (B, D) distances. Panels A and B show root-associated bacterial communities, while panels C and D include both root and soil communities. Colors indicate cultivation sites: Higashi-Kagawa (green), Ikenobe (red), and the university farm (purple). Symbols represent sample types and cultivars: ●, *Saccharum sinense* (S. sinense_1); ▲, *S. officinarum* (S. officinarum); ■, a commercial cultivar derived from *S. sinense* (S. sinense_2); and ◆, soil samples.
