# Supplementary material for: Root-associated Bacterial Communities of Sugarcane Cultivated for Traditional Wasanbon Sugar differ among Cultivation Sites in Kagawa, Japan
Source: Microbes Environ. 2026 Sep 4;41(3):ME26030. doi: 10.1264/jsme2.ME26030 (PMC13659341; doi:10.1264/jsme2.ME26030)
Supplement: Supplementary file 1 [file 41_26030_s1.pdf]

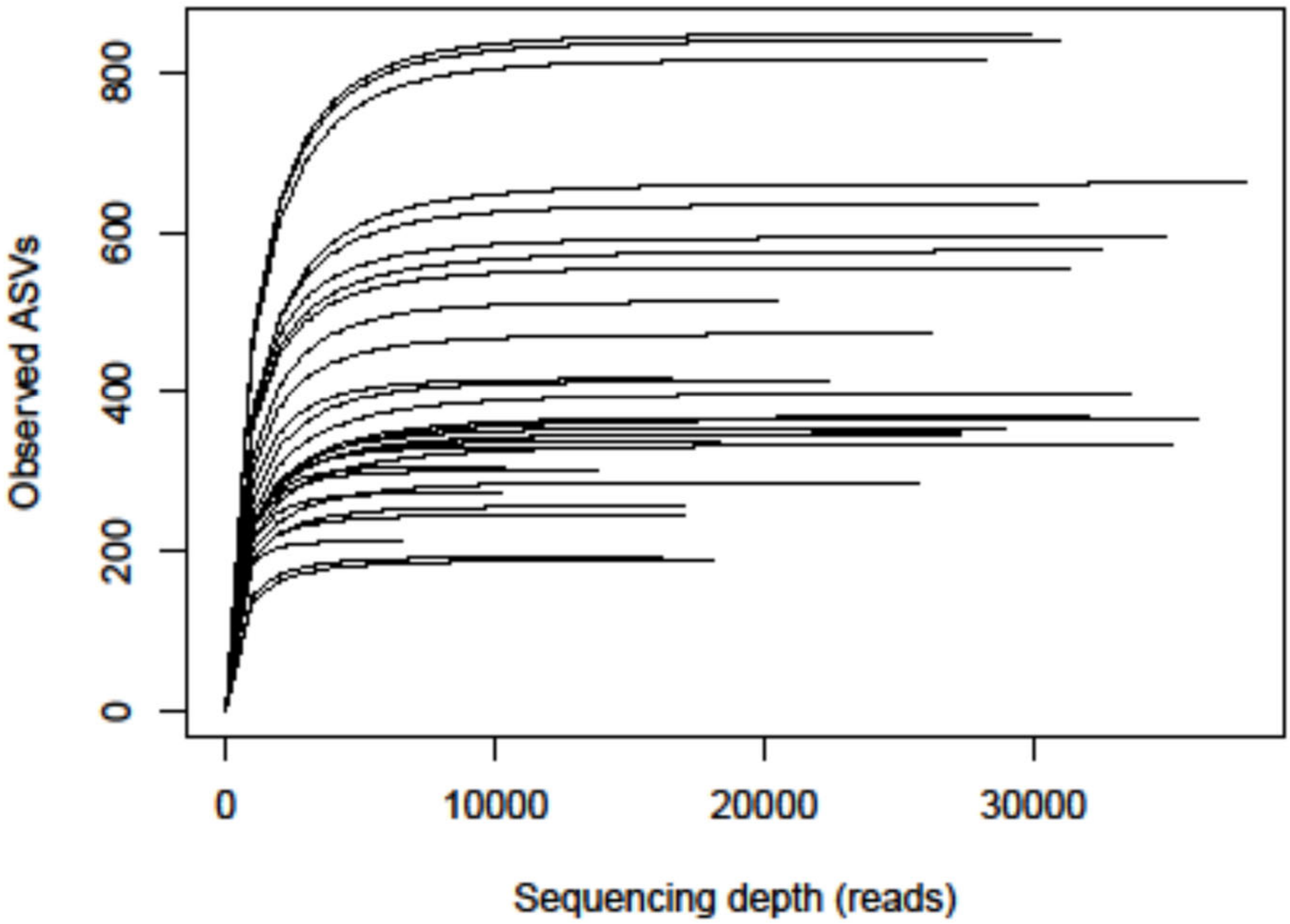

Fig. S1 Rarefaction curves of bacterial communities from root and soil samples based on observed ASVs. The curves approached saturation in all samples, indicating that the sequencing depth was sufficient to capture most bacterial diversity.

Supplementary Fig. S2., Yamada et al.,

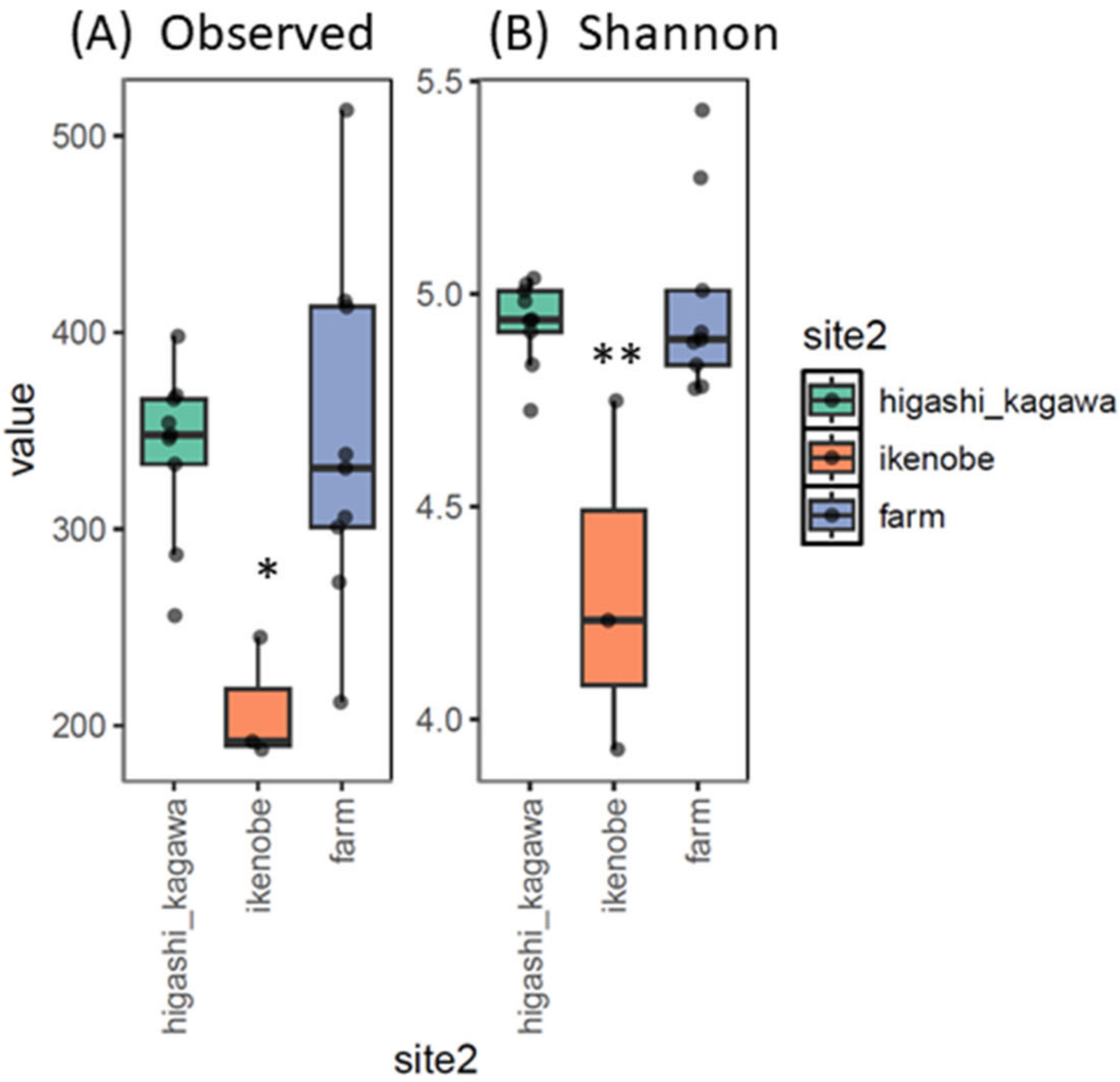

Fig. S2 Alpha diversity of root-associated bacterial communities among cultivation sites. Alpha diversity was assessed using observed ASVs (A) and the Shannon index (B). Asterisks indicate significant differences between Ikenobe and the university farm (*P < 0.01, **P < 0.001).

Supplementary Fig. S3., Yamada et al.,

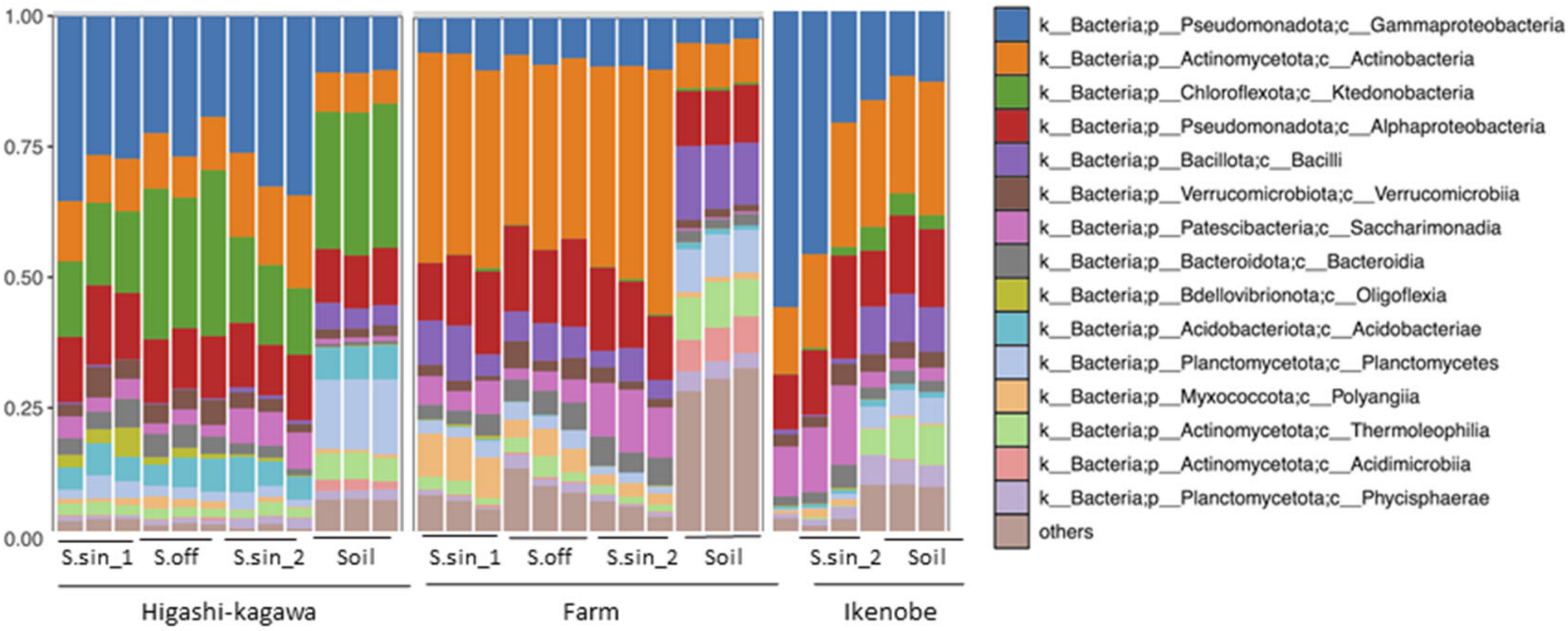

Fig. S3 Taxonomic composition of root-associated bacterial communities at the class level. Histograms show the relative abundances of the top 15 bacterial classes detected in sugarcane roots. Three cultivars, *S. sinense* (S. sin_1), *S. officinarum* (S. off), and a commercial cultivar derived from *S. sinense* (S. sin_2), were analyzed across three cultivation sites.

Table S1. Bacterial families significantly enriched in sugarcane root-associated bacterial communities at the Higashi-Kagawa site compared with the Farm and Ikenobe sites, identified by ANCOM-BC2 analysis.

taxon	log_fold_change	q_value
Ktedonobacteraceae	4.31	0.000002
Solirubrobacteraceae	2.91	0.000016
Acidobacteriaceae (Subgroup 1)	3.33	0.000036
Acetobacteraceae	2.81	0.000036
Burkholderiaceae	2.99	0.000118
Armatimonadaceae	2.59	0.000220
Isosphaeraceae	2.15	0.000349
Pedosphaeraceae	2.06	0.001440
Bryobacteraceae	1.92	0.001568
Unknown Family	1.92	0.002176
Rhodanobacteraceae	1.81	0.002176
Mycobacteriaceae	1.58	0.011710
Micropepsaceae	1.15	0.019640
Sphingobacteriaceae	1.35	0.019686
Caulobacteraceae	1.13	0.020442
Microbacteriaceae	1.27	0.039595
Polyangiaceae	1.27	0.042669
Alcaligenaceae	1.15	0.042669
Opitutaceae	1.17	0.042669
Beijerinckiaceae	1.09	0.044032

Table S2. Bacterial families significantly enriched in the Farm and Ikenobe root-associated bacterial communities identified by ANCOM-BC2 analysis

taxon	log_fold_change	q_value
Fibrobacteraceae	−3.56	0.00001
BIrii41	−2.72	0.00008
Steroidobacteraceae	−2.41	0.00022
Bacillaceae	−2.43	0.00157
Microscillaceae	−2.33	0.00157
Rhizobiaceae	−1.88	0.00218
Streptomycetaceae	−1.61	0.00346
Sphingomonadaceae	−1.31	0.00621
Micromonosporaceae	−2.11	0.00698
Saccharimonadaceae	−1.49	0.03250
Pseudonocardiaceae	−1.20	0.04403
